# Colicins and Microcins Produced by *Enterobacteriaceae*: Characterization, Mode of Action, and Putative Applications

**DOI:** 10.3390/ijerph191811825

**Published:** 2022-09-19

**Authors:** Katarina G. Marković, Mirjana Ž. Grujović, Maja G. Koraćević, Danijela D. Nikodijević, Milena G. Milutinović, Teresa Semedo-Lemsaddek, Milan D. Djilas

**Affiliations:** 1 Institute for Information Technologies, Department of Science, University of Kragujevac, Jovana Cvijića bb, 34000 Kragujevac, Serbia; 2 Innovation Center, University of Niš, 18000 Niš, Serbia; 3 Faculty of Medicine, Department of Pharmacy, University of Niš, 18000 Niš, Serbia; 4 Faculty of Science, Department of Biology and Ecology, University of Kragujevac, Radoja Domanovića 12, 34000 Kragujevac, Serbia; 5 CIISA—Centro de Investigação Interdisciplinar em Sanidade Animal, Faculdade de Medicina Veterinária, Universidade de Lisboa, Avenida da Universidade Técnica, 1300-477 Lisboa, Portugal; 6 Associate Laboratory for Animal and Veterinary Sciences (AL4AnimalS), 1300-477 Lisboa, Portugal; 7 Institute for Public Health of Vojvodina, Futoška 121, 21000 Novi Sad, Serbia

**Keywords:** *Enterobacteriaceae*, colicins, microcins, food biotechnology, medicine

## Abstract

*Enterobacteriaceae* are widely present in many environments related to humans, including the human body and the food that they consume, from both plant or animal origin. Hence, they are considered relevant members of the gastrointestinal tract microbiota. On the other hand, these bacteria are also recognized as putative pathogens, able to impair human health and, in food, they are considered indicators for the microbiological quality and hygiene status of a production process. Nevertheless, beneficial properties have also been associated with *Enterobacteriaceae*, such as the ability to synthesize peptides and proteins, which can have a role in the structure of microbial communities. Among these antimicrobial molecules, those with higher molecular mass are called colicins, while those with lower molecular mass are named microcins. In recent years, some studies show an emphasis on molecules that can help control the development of pathogens. However, not enough data are available on this subject, especially related to microcins. Hence, this review gathers and summarizes current knowledge on colicins and microcins, potential usage in the treatment of pathogen-associated diseases and cancer, as well as putative applications in food biotechnology.

## 1. Introduction

The family *Enterobacteriaceae* is a large, heterogeneous group of Gram-negative bacteria, which includes strains that naturally inhabit the gastrointestinal tract (GIT) of animals and humans. Being considered normal commensal members of the GIT microbiota, these microbes can also live and multiply in food environments [1]. Additionally, *Enterobacteriaceae* are acknowledged as indicators of food production hygiene, preservation, and storage, often being used as indicators of food quality and safety [1]. Additionally, *Salmonella* spp., *Yersinia enterocolitica*, pathogenic *Escherichia coli*, including *Escherichia coli* O157:H7, and *Shigella* spp., among others, are important foodborne pathogens [2]. Moreover, some members of this group, namely *Citrobacter*, *Enterobacter*, *Erwinia*, *Klebsiella*, *Kluyvera*, *Pantoea,* and *Serratia*, have been described both as harboring plant growth-promoting characteristics and attaining pathogenicity potential [3]. 

The taxonomic classification of *Enterobacteriaceae* was updated in 2016, when Adelou et al. [4] proposed a change based on phylogenetic analyses and molecular characteristics. The *Enterobacterales* order, which, until then, had a single *Enterobacteriaceae* family, now includes seven different families: *Enterobacteriaceae, Erwiniaceae* fam nov., *Pectobacteriaceae* fam. nov., *Yersiniaceae* fam. nov., *Hafniaceae* fam. nov., *Morganellaceae* fam. nov., and *Budviciaceae* fam. nov. The order *Enterobacterales* contains the type of genus *Enterobacter*, and its description coincides with the *Enterobacteriaceae* family [5].

According to the literature, the term bacteriocin is very often used for antibacterial peptides from Gram-positive bacteria, mainly produced by lactic acid bacteria—LAB [6]. Nevertheless, *Enterobacteriaceae* members also produce peptides and proteins, equally named bacteriocins, which have influence on microbial populations [7]. Bacteriocins produced by *Enterobacteriaceae* are generically called colicins. In fact, the first bacteriocin to be described in 1925 was produced by *Escherichia coli* [8]. Primarily known as “principle V”, it could have a limited influence on growth of another *E. coli* strain. This pioneering work has given impulse to many researchers to study antimicrobial (poly) peptides or proteins [7]. Moreover, many strains are able to produce two or more bacteriocins [9]. Colicins from *Enterobacteriaceae* and LAB bacteriocins have been studied a lot, in comparison with other bacteriocins, leading to a very high number of scientific reports on these antimicrobial substances [10,11]. In parallel with colicins, which are antimicrobial proteins with high molecular mass, lower molecular mass bacteriocins (microcins) have also been identified in *Enterobacteriaceae*, [12], but the studies and corresponding literature on these molecules are scarce.

Based on the aforementioned, this paper aims to perform a literature review on the importance of colicins and microcins produced by members of the *Enterobacteriaceae* family, and corresponding influence on prokaryotic and eukaryotic cells. Additionally, putative applications of these molecules on the treatment of pathogen-associated diseases and cancer therapy, as well as their potential application in food biotechnology, will also be addressed.

## 2. Colicins and Microcins 

**Figure 1:**
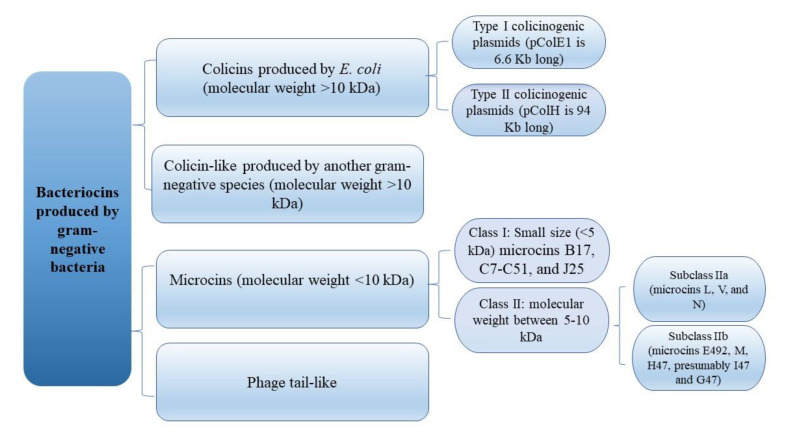
Classification of bacteriocins produced by Gram-negative bacteria.

Bacterial resistance to available antibiotics is a huge problem, and that is why there is great interest in available natural alternatives, such as the use of bacteriocins. [13,14]. Bacteriocins produced by *Enterobacteriaceae* are ribosome-synthesized proteins, known as colicins and microcins [15]. Colicins are typically large proteins of high molecular mass, while microcins have low molecular weight [16,17] (Figure 1). *Enterobacteriaceae* often produce bacteriocins under stress conditions, such as the SOS response system [9]. Colicins and microcins can inhibit the growth of competing *E. coli* strains and other phylogenetically related bacteria [17]. A wide array of colicins and microcins use numerous cytotoxic mechanisms as mode of action, including pore formation, degradation of peptidoglycan precursors, phosphatase activity, RNAse activity (often targeting 16S rRNA and specific tRNAs), and DNAse activity [17]. Co-synthesizing specific immunity protein from producers protect producers from self-killing during bacteriocin antibacterial action [18,19].

### 2.1. Colicins—Short Overview of Genetic Organization, Classification, and Mechanisms of Action

Colicins are the most investigated group of bacteriocins. These molecules presenting a molecular mass 30–80 kDa are produced by specific *E. coli* strains and act against another *E. coli* strain and other *Enterobacteriaceae* [7,17,20,21,22,23]. Colicin-producing *E. coli* harbor colicinogenic plasmids, generically named pCol [7]. There are two types of colicinogenic plasmids, differentiated by size, number of copies in the cell, amplification ability, and aptitude to be transferred by conjugation. Type I colicinogenic plasmids are small (for example, pColE1 is 6.6 Kb), occur in a large number of copies in the cell, and encode group A colicins. Type II colicinogenic plasmids are large in size (e.g., pColH is 94 Kb), only one copy per cell, and are often capable of transfer by conjugation [24]. Large plasmids can contain either one or two colicin operons; therefore, the cells containing type II plasmids may be able to produce up to two distinct colicins. Various plasmids can encode similar colicin proteins [10,17,25]. Colicin’s operons involve a gene cluster consisting of two to three genes: structural gene (*col* or *cxa*), the gene for immunity protein (*imm* or *cxi*), and the gene for the lytic protein (*kil* or *cxl*) [26]. The gene encoding the immunity protein is located downstream from the structural gene. Operons encoding ionophoric colicins do not include an immunity gene, but it is found on an opposite strand and has its own promoter. Genes encoding the lysis protein are always the last gene in the operon of group A colicins, while members of group B usually do not possess a lysis gene. The product of *cxl* is a lysis protein (also called colicin release protein or bacteriocin release protein), a small lipoprotein involved in the release of colicin into the medium and cell death after induction. Some functional regions of colicin genetic systems encode products associated with tissue adherence, serum resistance, and ability to conjugate [10].

Colicins are not produced in bacterial cells under normal conditions, but a limited number of colicin molecules are constantly present in the cell. Colicin amount drastically increases under the effect of various DNA-damaging agents (e.g., UV light and antibiotic mitomycin C) and environmental factors, such as the lack of nutrients or increased bacterial population density [27]. Agents that cause DNA damage or stress lead to an “SOS response”, activating RecA proteinase, followed by auto-cleavage inactivation of LexA protein (repressor of colicin synthesis), hence allowing the transcription of the colicin operon [28]. In addition, alternative mechanisms of colicin synthesis activation, such as thymine starvation, catabolite repression, and mutation of *ompR* gene, have been described [29,30]. Expressed colicins accumulate in the cytoplasm of the producing cell and need a significant amount of colicin lysis protein to be released to the extracellular medium. Colicin lysis proteins are small lipoproteins that show a high level of similarity in genetic sequence (*cxl* gene) and are co-expressed with colicin protein. The role of colicin lysis allows colicin release by provoking quasilysis, which causes structural changes and lysis of the cell envelope, activation of outer membrane phospholipase A, and death of the producing bacterial cell. Type II plasmids of group B colicin do not consist of a lysis gene (colicin D is an exception); therefore, their synthesis is not lethal for the producer cell [17,31].

Colicins show similar types of structural organization, including three distinct domains: one domain that is involved in the recognition of specific receptors, another domain with a role in translocation, and a third domain involved in the lethal activity. This organization is in coherency with the mechanism of action, in the sense that each phase of colicin action involves one domain of the colicin molecule [32]. The process of interaction between colicin and the cell of interest involves two or three steps. The first step includes binding to a specific receptor on the cell surface, followed by translocation of the colicin molecules across the cell envelope, followed by the action that causes bacterial death during the third step. The recognition step involves several receptors that are commonly involved in the uptake of essential nutrients (e.g., siderophore-bound iron, vitamin B12, or nucleosides). These receptors are parasitized by colicins, helping them enter more efficiently in the target bacteria [7]. The translocation step involves one of two distinct systems—Tol and Ton, on which the classification of colicins to groups A and B is based, respectively [33]. Colicins A, E1-E9, K, L, N, S4, U, and Y belong to group A. Colicins B, D, H, Ia, Ib, and M belong to group B. Tol system is formed by TolA, TolQ, TolR, and TolB, as well as Pal protein. Genes for proteins of translocation systems are found in clusters on *E. coli* chromosomes. The Ton system is formed by three inner membrane proteins (e.g., TonB, ExbB, and ExbD). Colicins of group A require all proteins from the Tol system or a subset of them, while all proteins from the Ton system are necessary for translocation of group B colicins [33,34,35].

Colicins from other close related bacteria (e.g., klebicins produced by *Klebsiella* spp. or S-pyocins produced by *P. aeruginosa*) have a similar size, structure, and function to *E. coli* bacteriocins. Similar to colicins, their antimicrobial activity can be due to pore formation or nuclease activity [36]. Inside each of the two groups, colicins and colicin-like bacteriocins may be differentiated according to their bactericidal mechanisms [21]. Colicins kill target cells through three different mechanisms: (a) by making voltage-dependent channels in the inner membrane of the target bacteria, (b) by a nuclease action in the cytoplasm (DNase, 16S rRNase, and tRNase activities), or (c) by inhibiting peptidoglycan synthesis [7,12,17,37]. In further detail, colicins undergo a series of conformational changes during the voyage from the extracellular space to the periplasmic membrane of the target cell. All channel-forming colicins act in a similar way. The receptor domain of the molecule mediates the first interaction with the host cell by recognizing membrane proteins, such as BtuB (B12 receptor) or iron transport proteins. The pore-forming domain of the colicin molecule (C-terminal domain) is made of a tightly packed group of 10 α-helices. Studies show that this process involves initial fast absorption to the membrane of the target cell, followed by slow insertion of C8 and C9 helices into the membrane interior. Helices C8 and C9 form the hairpin structure, but these changes happen after interaction with the inner membrane [38]. The process of colicin insertion and pore formation does not involve target cell proteins [39]. Several models of a closed channel have been proposed [40,41]. The positive voltage applied to the closed channel leads to further insertion into the membrane pore. Furthermore, only several helices are necessary to form a viable pore [42]. All colicin channels are voltage-dependent and present high selectivity for protons over other cations, opening at the positive voltage and closing at the negative voltage. There are multiple open and closed states of the pores [43,44]. The cell-killing potential of pore-forming colicins is extremely high, as one colicin molecule is enough to destroy a target cell. Immunity proteins, encoded by the same plasmids as the colicin, are small polypeptides that act as a protection system against self-produced colicin and exogenous colicins secreted by neighboring bacterial cells [17]. 

Colicins with enzymatic activity may act as hydrolases or transferases that target phosphodiester bonds in the DNA (DNase) or RNA (rRNase or tRNase) of the host cell. Colicins begin their passage into the target cell via Ton or Tol systems, translocate through the inner membrane, and enter the cytoplasm, where they exert the lethal activity. DNase colicins degrade DNA by producing dents in dsDNA by repeated cleavage [17]. These colicins are metal-dependent but the nature of the metal ions required is still unknown, although research suggests that Zn^2+^ and Ni^2+^ are necessary as cofactors for enzyme activity [45,46]. RNase colicins may cause cell death by inhibiting protein synthesis and, for this activity, no cofactors are required. Target molecules of RNase colicins are 16S rRNA and anticodon loops of tRNA [17]. Nuclease colicins are produced and released from the cell in complex with its nuclease-specific immunity proteins, which protect the producing cell from the lethal activity [47].

Colicin M, firstly described in 1974, presents a distinct mode of action, since it is released without immunity protein and causes the death of the host cell by inhibiting peptidoglycan synthesis, specifically by degrading peptidoglycan precursors, and LPS O-antigen production. Colicin M activities can be inhibited by changing the osmolarity of the growth medium [11,23,48,49,50].

### 2.2. Microcins—Short Overview of Genetic Organization, Classification, and Mechanisms of Action

Microcins are low molecular mass bacteriocins (ranging from 1 to 10 kDa), produced by Gram-negative bacteria, often *E. coli*, under stress conditions [51,52], which respond very well to changes in pH, protease activity, or temperature alterations. Microcin’s classification considers three criteria: (a) the presence, nature, and localization of post-translational modifications, (b) gene cluster organization, and (c) leader peptides sequences, therefore, separating microcins into two classes—class I and II [7]. 

Class I microcins are low molecular mass peptides (below 5 kDa), namely microcins B17, C7–C51, and J25. They are plasmid-encoded and go through post-translational modifications [53,54]. Class II includes peptides of higher molecular mass (in the range between 5 and 10 kDa), and is further divided into subclasses IIa and IIb. Class IIa contains plasmid-encoded peptides that do not undergo post-translational modification and are possibly forming disulfide bonds (e.g., microcins L, V, and N). Class IIb includes chromosome-encoded linear microcins, which may carry a C-terminal siderophore post-translational modification (e.g., microcins E492, M, H47, and, presumably, I47 and G47) [55,56,57].

Hence, microcins are encoded by gene clusters located either in plasmids or by the bacterial chromosome. Gene clusters involved in microcin production include a variable number of determinants [7], with minimal structure organization, consisting of a structural gene, the self-immunity gene, and genes encoding the export system. For class I microcins, the self-immunity gene is not located near the structural gene, while genes involved in the post-translational modification are adjacent to the structural gene. In addition, at least one gene is involved in both secretion and self-immunity. A gene cluster of class I microcin B17 is located on single-copy plasmids found in *E. coli* and consists in seven genes forming an operon: *mcbA* encodes the B17 precursor, *mcbB*, *mcbC*, and *mcbD* encode components for post-translational modifications of McbA, while *mcbE* and *mcbF* have roles in secretion and self-immunity, and *mcbG* is required for full self-immunity. Microcin C (C7–C51) is the smallest microcin, characterized in *E. coli* strains and also located on a single-copy plasmid. The corresponding gene cluster is composed by *mccA*, *mccB*, *mccC*, *mccD*, *mccE*, and *mccF*, found only on the C51 genetic system. Structural gene *mccA* is only 24 bp in length, making it one of the shortest genes known. The last gene *mccF*, transcribed from the opposite strand, has a role in self-immunity towards C7, but not in self-immunity towards C51. Maturation of microcin C does not take place in the producing cell but in target cells after entrance through OmpF and Yej channels. Microcin J25 is found on low-copy-number pTUC100 plasmid in *E. coli* strains and its genetic structure consists in four genes (*mcjA*, *mcjB*, *mcjC*, and *mcjD*), organized in two operons. Gene *mcfA* encodes a precursor that must undergo modification by proteins encoded by *mcjB* and *mcjC* to form the distinct lasso structure [58,59,60,61]. 

As previously mentioned, class II microcins are divided into two subclasses, IIa and Iib. A set of genes that have a role in peptide export (at least two) are homologous between subclasses and require *tolC* to be functional. Class Iia microcins possess four genes organized in a similar way, all of them located on a plasmid. Genetic organization of microcin L, produced by *E. coli* LR05 strain involves genes *mclC*, *mclI*, *mclA*, and *mclB*. Gene *mclC* encodes peptide precursor, *mclI* is involved in self-immunity, while *mclA* and *mclB* have a role in peptide export [62]. Subclass IIb microcin genes are chromosomally encoded and show a complex transcriptional organization. Microcin E492 is the most well-studied, being secreted by *Klebsiella pneumoniae* RYC492, and its gene cluster is composed of 10 genes (*mceA* to *mceJ*) organized in six transcriptional units that are crucial for production, export, and self-immunity [52,61].

Microcin gene clusters have lower G + C content in comparison to their bacterial host genomes and are flanked by direct repeats, which could suggest that producing bacteria are not their original host and highlights the possibility of horizontal gene transfer events. As an example, the G + C content of microcins C7 and C51 gene clusters is 34%, which is significantly lower than 51%, found in the host *E. coli* chromosome [63].

Microcins production is up-regulated when bacteria are exposed to stress conditions, such as the lack of nutrients, oxygen or nitrogen starvation, iron availability in the culture media, and mild air limitations, among others. In many cases, variation in pH values and cell density does not affect microcin production. These molecules are produced as inactive precursor peptides with a core structural sequence and an N-terminal leader peptide (microcin C is an exception, as it does not possess a leader peptide). Leader peptides have various functions in different microcins, and previous data suggest their role in the protection of the precursor against degradation, keeping the precursor inactive inside the producing cell, and that cleavage of the leader peptide at a specific cleavage site allows maturation and secretion of the microcin molecule [52,53]. Microcin maturation requires proteolytic enzymes that cleave the leader peptides and enzymes that ensure post-translational modifications. Class I microcins use a variety of different mechanisms for export, which includes efflux pumps or systems similar to ABC transporters. The processing and maturation of microcin MB17 are carried out by chromosomally encoded enzymes, mostly by metalloprotease PmbA, while export of microcin out of the cell is carried out by an ABC-type transporter McbEF. Experiments showed that the export machinery for B17 is not involved in the cleavage of the leader peptide [64]. Proteolytic cleavage of class II microcin leader peptide occurs during export. Secretion and maturation of class II microcins from the producing cell usually involve the machinery that includes the ABC transporter, its accessory proteins, and TolC protein [56]. Some microcins enter the bacteria as harmless molecules and become toxic after the maturation process occurs inside the target cell [54]. A large range of different post-translational modifications of microcin molecules involve enzymes encoded on the microcin gene clusters. At least three genes are involved in post-translational modifications of B17 microcin [52]. Microcins have receptor-mediated mechanisms of antimicrobial activity, as they overtake receptors that have an important role in nutrient uptake. Receptor types range from siderophore receptors (for microcins J25, L, M, V, and others) or porin OmpF (in the case of microcin B17). Translocation of the microcin protein requires protein complexes of the inner membrane, such as TonB or different ABC transporters [65,66].

Once inside the target cell, microcins present distinct and complex mechanisms of action. Some mechanisms of action have been studied in detail, while others remain to be confirmed [12]. Some microcins act by forming pores in the bacterial membrane, inhibiting aspartyl-tRNA synthetase and DNA gyrase GyrB. Other mechanisms include inhibition of secondary RNA polymerase channel, preventing transcription and inhibiting cellular respiration (J25), impairing the cellular proton channel (e.g., H47 and probably M and I), or the ATP synthase (H47) [12]. Class I microcins inhibit the activity of essential enzymes, such as DNA gyrase necessary for DNA supercoiling. B17 inhibits DNA supercoiling and induces the accumulation of complexes of DNA gyrase and cleaved DNA, leading to the formation of breaks in dsDNA and inhibition of DNA replication [67]. Other microcins, such as J25, target RNA polymerase, obstructing it in the active site and blocking the transcription process [68]. Microcin J25 also influences mitochondria and the respiratory chain [52]. Class II microcins target the inner membrane of the cell of interest and require proteins of the inner membrane for their activity. For example, microcin H47 targets the F_0_ proton channel of ATP synthase [69]. Microcin V needs protein involved in serine uptake to permeabilize the inner membrane [54].

Certain high-molecular-weight peptides present cylindrical structures, which are highly similar to phage tail structure, can perforate the bacterial cell membrane and lead to cell death [70]. These antimicrobial peptides are named phage tail-like bacteriocins, derived from the domestication of phage tail genes (i.e., genes for peptide release and regulatory genes). The most well-studied bacteriocins from this group are R-pyrocuns and F-pyocins, produced by *Pseudomonas aeruginosa*. Their mechanism of action is the disruption of the membrane potential leading to pore formation on the bacterial membrane [36,71].

## 3. Interaction of Colicins and Microcins with Prokaryotic and Eukaryotic Cells

Interactions are probably the most frequent processes occurring in the microbial gut, which are influenced by intestinal microniches [12]. Most likely, the diversity and stability of the intestinal microbiota depend on competitive interactions [72]. All members of the microbial gut compete for the same energy, nutrients, surfaces suitable for adhesion, etc. Competition reduces the reproductive capabilities of competitors (exploitative competition) [73]; however, this antagonistic interaction rarely leads to the extinction of competitors. Fluctuations/variations in the environment may favor this competitor in other, sometimes immediate, circumstances. In the GIT, the competition between bacterial species is well represented and based on the fight for nutrients, where, in the process, they kill or inhibit the growth of each other, which is known as allelopathy or amensalism [74]. 

### 3.1. Colicins

Certain strains of colicin-producer *E. coli* are able to inhibit the Shiga-toxin-producing *E. coli*, a pathogenic strain able to cause disease in humans [75]. Moreover, during in vitro experiments conducted by Stahl et al. [76], colicin E1 and colicin N presented an inhibitory effect on enterotoxigenic *E. coli*. 

Today, many bacteria show resistance to antibiotics. According to Brown et al. [77], colicin-like bacteriocins are highly effective at killing target cells growing in biofilm state and may constitute useful therapeutic options for the treatment of chronic bacterial infections. Trautner et al. [78] have shown that pre-growth of colicin-producing *E. coli* K-12 on catheters can prevent colonization by a colicin-susceptible *E. coli* clinical isolate, indicating that colicin production may act as a potent inhibitor of biofilm formation [79].

A novel colicin type, colicin Z (26.3 kDa), is generated by an original producer, extraintestinal *E. coli* B1356, isolated from the anorectal abscess of a 17-year-old man. Colicin Z has a limited inhibitory spectrum, being active only against enteroinvasive *E. coli* and *Shigella* strains. Moreover, in a recent study, all *E. coli* and *Shigella* isolated between the years 1958 and 2010 showed sensitivity to colicin Z. The lethal effect of colicin Z is associated with the degradation of the cell wall peptidoglycan [80].

Furthermore, Tahamtan et al. [81] indicated that the use of colicin and biotherapy, instead of antibiotics, may be more efficient for the control of *E. coli* K99 infection (a strain recovered from cattle gastrointestinal tract). Colicin supplementation protected treated mice and inhibited *E. coli* K99 colonization. Hence, the usage of colicins in animal farms should be considered a valid option.

### 3.2. Microcins

Microcins are antibacterial substances, which exhibit a wide range of post-translational modifications. They originate exclusively from enterobacteria, mainly *E. coli*, and show antibacterial activity against Gram-negative microbiota [52]. Majeed et al. [82] suggested a high distribution and diversity of microcins in *E. coli*, compared to other *Enterobacterales*. This is probably a consequence of a highly competing lifestyle inside intestinal subniches. Bacterial species that live in less or more specific niches (*Shigella* spp. or *Salmonella* spp.) are less microcinogenic. In addition, the genera *Citrobacter*, *Klebsiella*, and *Enterobacter*, which have wider environmental lifestyle [83], contain characteristic microcins compared to *E. coli*.

In class I microcins, the most well studied are microcin B17 and microcin J25 [12]. The majority of the research on Microcin B17 (MccB17) has been study with *E. coli* strains. However, there are many studies of MccB17-like activities in environmental *Pseudomonas* spp. (*P. syringae* and *P. antarctica*) [84,85]. Microcin J25 (MccJ25) is active against *Salmonella* species and *E. coli* [86]. In class IIa, the most investigated microcins are microcin V (MccV), microcin L (MccL), microcin N (MccN), and microcin S (MccS). Microcin V showed inhibitory effect on the bacteria from the genera *Klebsiella*, *Escherichia*, *Salmonella*, and *Shigella* [87]. Microcin L, produced by *E. coli* LR05, acts against related *Enterobacteriaceae*, including the *Salmonella enterica* serovars Typhimurium and Enteritidis [88]. Other authors indicated that Microcin N showed inhibitory activity against *E. coli* and *S.* Typhimurium, but were not active against *Listeria monocytogenes* or *Campylobacter jejuni* [89]. It is known that microcin S inhibits the growth of enterohemorrhagic and enteropathogenic *E*. *coli* [90,91]. In class IIb, most studies regard microcin E492 (MccE492), microcin H47 (MccH47), microcin M (MccM) [56], and microcin N (MccN), which is also recognized as microcin 24 [92]. Antimicrobial activities of other mentioned microcins from class IIb are restricted to some species of *Enterobacteriaceae* [12].

One of the functions attributed to microcins is the establishment of interactions with eukaryotic cells. Microcins are able to cross the intestinal–blood barrier and cause a systemic effect on the host [93]. MccJ25 microcins, interact with integrins, receptors from eukaryotic transmembrane, and potentially regulate the cell cycle [94]. Bacteriocins from *Enterobacteriaceae* may show genotoxicity, which is bad for the host. Genotoxic effects of bacteriocin from *Citrobacter freundii* were detected on mice bone marrow cells in vivo and concentrations of 150 and 300 mg/kg caused an increase in the micronuclei frequency in bone marrow cells. Furthermore, DNA damage increased significantly and proportionally to higher bacteriocin doses [95].

The host’s immune system has significant role in the growth of pathogens. It modulates the balance between desirable microbes and pathogens and regulates gut microbiota [96]. Some authors propose that the gut environment plays a crucial role in generating conditions for bacterial competition by colicin Ib (ColIb) [97]. The environmental conditions in inflammation-inflicted blooms favor colicin-dependent competition of *Enterobacteriaceae* by triggering ColIb production and susceptibility at the same time. Thus, the authors reported the role of colicins as important bacterial factors in inflammation-induced blooms [97]. The immunomodulatory effect of enterobacteria and their bacteriocins has been investigated [98,99]. According to Pang et al. [98], gut microbiota, including *E. coli*, suppresses allergic responses in mice and allergic airway disease. *E. coli* infections induced attenuated allergic response, reduced eosinophil inflammation, decreased serum level of IgE, and reduced IL-4 cytokine. Another study showed the immunomodulatory effects of Pheromonicin-NM (polymorphonuclear leukocytes-NM), the new engineered bactericidal peptide (colicin Ia and an anti-porin A antibody mimetic) [99]. PMC-NM induced innate immune responses of bovine mammary epithelial cells infected by *E. coli* via higher mRNA expression of TLR2, IL-1β, IL-8, lactoferrin, LAP, TAP, and DEFB1. This study further suggests PMC-NM as an ideal antibacterial agent against *E. coli*, which causes mastitis.

## 4. Potential Application of Colicins and Microcins 

So far, there are a limited number of investigations of the potential application of colicins and microcins, and bacteria that produce them. However, there is a huge potential and need for investigation in this field. The present manuscript discusses information regarding some previous reports related with the application of colicins and microcins in distinct areas. 

### 4.1. Applications in Medicine

#### 4.1.1. Antimicrobial Activity 

Colicins and microcins are antibiotic peptides responsible for blocking vital functions in the target cell. Colicins, larger polypeptides, mainly act by pore formation, nuclease activity, and inhibition of peptidoglycan synthesis (e.g., colicin M). Microcins act by forming pores in the bacterial membrane; by inhibiting aspartyl tRNA synthetase and/or DNA gyrase and causing double DNA breakage. Colicins and microcins production includes self-protection (immunity) involving acetyltransferases, the production of immunity proteins (that belongs to Class IIb microcins), efflux pumps, or inhibition of DNA gyrase supercoiling activity, among others. The influence of peptides or their derivatives, released from the producer cell, that possesses antibacterial activity frequently depends on membrane receptors present in the target cells. Some microcins use the “Trojan horse” strategy by mimicking essential nutrients (such as essential amino acids or iron-siderophores) to be incorporated into the target cell [12]. Immunity could explain the absence of “suicide” in the producing strains, while resistance means acquired insensitivity to external microcins. Mutations are the basis for most microcin resistance mechanisms. Surely, mutational resistance may develop in microcin-sensitive bacteria during the amensalistic–competitive interactions between them. Mechanisms of immunity and resistance to microcins have been described in detail by Baquero et al. [12]. 

The negative impact of *Enterobacteriaceae* on the host’s immunity is the appearance of Crohn disease. This chronic syndrome is caused by bacterial dysbiosis that frequently involves colonization by adherent-invasive *E. coli,* which has the ability to form biofilm, invade host cells, and stimulate the production of proinflammatory cytokines. Colicins show potent activity against the *E. coli* biofilm. Colicins E1 and E9 can kill *E. coli* but showed no toxicity towards macrophage cells or stimulating the production of proinflammatory cytokines [100]. According to Nedialkova et al. [97], gut inflammation favors the overgrowth of *Enterobacteriaceae*. These authors showed that a pathogenic *S.* Typhimurium has benefited from ColIb (colicin by *Salmonella*) production in competition against commensal *E. coli*. In the absence of gut inflammation, ColIb production did not confer a competitive advantage to *S.* Typhimurium. These findings reveal the role of colicins as important bacterial proteins in gut inflammation processes.

#### 4.1.2. Cancer Therapy

Cancer is undoubtedly one of the leading health problems, with a growing incidence in the last years [101]. The conventional use of chemotherapeutics in anticancer therapy is unsatisfactory because many of the molecules applied show serious side effects due to their nonselective cytotoxicity, as well as to the development of drug resistance [102,103,104]. Thus, the search for alternative treatments is a necessity and it has become the hotspot of global science and media attention. There is an increasing number of research and attempts for discovering, isolating, or synthetizing anticancer substances with improved properties, compared to currently used chemotherapeutics. It has been reported that natural substances may show selective anticancer activity, induce apoptosis in cancer cells, and provoke lower adverse effects on healthy cells. Among them, secondary metabolites produced by plants or fungi constitute a relevant source of promising anticancer agents [105,106,107,108,109]. Additionally, it has been reported that proteins from other natural sources, such as animal venoms or protein structure such as fibroin and sericin from insects’ silk, also show selective anticancer and proapoptotic activity [110,111,112]. Another promising area of research involves the study of therapeutic peptides and proteins, including bacteriocins, produced by bacteria, due to their ability to affect cancer cells [113,114,115].

Primary, *colicins* are interesting due to their effects on prokaryotic cells and probiotic properties [10,12]. Some previous studies indicate the therapeutic potential of bacteriocins produced by various bacteria against cancer cell lines [113,114]. Among them, it has been frequently reported that colicins show selective effects in anticancer activity [116,117,118,119]. This selectivity towards cancer cells is particularly relevant regarding experimental and clinical trials. Among the first investigations on the anticancer properties of colicins, colicin 3 inhibited the proliferation of P338 murine leukemia cells, without effects on normal cells [120]. It has also been reported that different colicins (e.g., A, E1, U, and E3) induce cytotoxic and proapoptotic activity on breast cancer cell lines (e.g., BT474, BT549, MDA-MB-231, SKBR3, and T47D), colon cancer (HT-29), osteosarcoma (HOS), leiomyosarcoma (SKUT-1), and fibrosarcoma (HS913T) [117]. HT-29, known to be a highly resistant cell line, was resistant to colicin E1, while colicin E3 inhibited the growth of all tested cells. 

Although there are several reports confirming colicins’ cytotoxic activity on cancer cell lines, to our knowledge there are only a few studies that deal with the molecular mechanisms behind this activity. Hence, cytotoxicity may be direct, by changes on cytoplasmatic membrane permeability by pore-formatting mechanisms and through the induction of apoptosis on human cancer cells [117]. It is well known that the release of some apoptotic activating signals is a common event after a change in mitochondrial membrane permeability [121]. These events lead to apoptosis, which was also confirmed in the study of Chumchalová and Smarda [117]. Proapoptotic activity of colicins is favorable, since it may lead to the elimination of malignity-transformed cells in the digestive tract microenvironment. Apoptosis can be triggered by different mechanisms, including the activation of death receptors on the cell membrane and signals out of the cell, by activation of external apoptosis pathway, or include stressors in the cells, main signals from mitochondria, and DNA damage that start the internal apoptosis pathway [110,122]. Mechanisms of apoptosis induced by colicins have been scarcely investigated. Colicin N, as representative of the pore-forming colicins, induces proapoptotic activity on lung cancer cells (H292 and H23). This study reports that colicin N reduces caspase 8 inhibitors (c-FLIP) and antiapoptotic protein Mcl-1, as well as the inhibition of Integrin/Akt signaling responsible for cell survival and avoidance of apoptosis [123]. Colicins also show nonspecific DNase activity, a highly specific RNase activity [118]. 

Colon cancer is often preceded by inflammation, obstructive disease, or impaired pH in the intestine [124]. Prophylaxis implies, among other things, lifestyle changes and diet aiming toward the suppression of frequent inflammation and disturbances of the intestinal microbiota [125]. Occurrence and pathogenesis of colon cancer are related to the absence of colicinogenic strains in the intestine, which indicate an impact of colicins on the prevention and suppression of cancer progression [118]. An in vivo study with 30 colon cancer patients reported that individuals with aggressive forms of cancer harbor more virulent *E. coli* strains, with an advent of the higher production of colicins on their mucosa [126]. The same authors also reported a study with 63 patients, which analyzed the production of colicins and microcins in different neoplasia’s in the colon, namely advanced colon adenoma and colorectal carcinoma. It has been shown that the highest production of colicins occurred in patients with colorectal carcinoma, while microcins were produced in the largest percentage in individuals with advanced adenoma. Differences in colicin production between sexes were also reported; men with colorectal carcinoma produced a significantly higher amount of colicins and microcins compared to women with the same type of cancer, while women with advanced adenoma produce a significantly higher amount of microcins compared to men [127].

Due to the close connection between inflammation and the promotion of colon cancer, colicins have also been considered potential antibiotics with a positive effect on maintaining the balance within gut microbiota, especially in Crohn’s disease [77], inflammatory bowel disease and dysregulation of the mucosal immune system of the gut leading to uncontrolled inflammation, associated with colorectal cancer [128]. Colicins also have in vivo beneficial effects on the murine model system, by alleviating the symptoms of gastrointestinal infections [129]. Additionally, an in vivo study showed protective anticancer effects of E3 colicin by decreasing the tumor mass in mice, after subcutaneous application [10].

*Microcins* have the ability to interact and influence eukaryotic cells. Previous studies indicate their antitumor properties [130,131] and ability to interact with eukaryotic transmembrane proteins [94]. Microcin E492 has shown cytotoxic activity on Jurkat and HeLa cell lines, as well as on T cells in acute leukemia [131,132]. Cytotoxic activity being associated with apoptosis in lower concentrations and necrosis in higher amounts. Other reports also indicate that microcins induce morphological and biochemical changes characteristic of apoptosis, including cell shrinkage, phosphatidylserine release, DNA fragmentation and condensation, the release of intracellular calcium ions, loss of mitochondria membrane permeability, and the activation of caspases, among others [132]. Microcin MccJ25 has shown cytotoxic and proapoptotic activity on MCF-7 and MDA-MB-435 multidrug-resistant breast cancer cells [133]. Microcin B17 showed the ability to prevent replication, derived from DNA gyrase inhibition [134]. Earlier studies show that microcin E492 induces direct cytotoxicity on cancer cells by pore formation and disruption of membrane potential; [132,135] have demonstrated the effects of microcin E492 on two human colorectal cancer cell lines in vitro, HT29 and SW620 cells. Moreover, these cell lines were used for in vivo transplantation and developing zebrafish xenograft models; results have shown that HT29 transplantation was less successful. The efficacious transplantation of SW620 cells showed that the injection of microcins in the xenograft reduced tumor size [135]. Additionally, the anticancer potential of microcin E492 also has been shown in human colorectal carcinoma xenografts grown in nude mice [131]. 

## 5. Applications in Food Biotechnology

### 5.1. Antimicrobial Activity 

As previously mentioned, bacteriocins produced by *Enterobacteriaceae* can be used to control the development of foodborne pathogens, such as *E. coli* O157:H7, *Shigella* spp. (e.g., *S. dysenteriae, S. flexneri, S. boydii,* and *S. sonne*), and *Salmonella* spp. (e.g., *S. typhi* and *S. typhimurium*), among others. The colicin may be applied to dairy products and/or onto hard surfaces, such as the surfaces of meat processing equipment and other related apparatus, such as a cutting board, to inhibit the growth of pathogenic organisms. The colicin may affect the inhibition of the growth of a pathogenic *Enterobacteriaceae*, preferably *Escherichia coli* strain O157:H7. The colicin may be formulated as a liquid composition, in admixture white water, or as a dry form, such as a freeze-dried, powder (concentrate or diluted), and the like. The invention also gives a method for inhibiting pathogenic *Enterobacteriaceae* on and/or in a food product [136]. According to Patton et al. [137], colicins from Gram-negative bacteria may be used against foodborne pathogens. ColE1 was produced from an *E. coli* K-12 strain containing plasmid pColE1-K53. ColE1 effectively reduced populations of *L. monocytogenes* in broth culture and on meat product surfaces. ColE1 significantly inhibited the growth of *L. monocytogenes* for up to 3 days. An understanding of this mechanism of action could lead to broader applications of ColE1 against many other bacterial pathogens. ColE1 is a safe and highly effective anti-*Listeria* agent. Divercin AS7 is a class IIa bacteriocin produced by *Carnobacterium divergens* AS7. It exhibits antibacterial activity against pathogens and food spoilage flora, including *Listeria* spp. The safe use of bacteriocins as food biopreservatives requires the absence of cytotoxicity in human cells. To analyze the effect of divercin AS7 on human enterocytes, recombinant divercin AS7 was expressed in *E. coli* strain BL21DE3pLis and in vitro studies were performed to evaluate the safety of recombinant divercin AS7. No cytotoxic effect on differentiated monolayer Caco-2 cells and apoptotic appearance was observed when recombinant divercin AS7 was used at a concentration of 2 μg/mL. In a study by Olejnik-Schmidt et al. [138], divercin AS7 did not interfere with the monolayer integrity of differentiated Caco-2 cells. The obtained results suggest that divercin AS7 is a promising peptide for the food industhis. ry. In 2015 and 2017, Nomad Bioscience GmbH applied to the Food and Drug Agency (FDA) to approve the use of their colicin as a GRAS antimicrobial agent for the control of *E. coli* in fruit, vegetables, and meat. The FDA has approved the potential use of colicin in the food industry, through careful consideration of all safety aspects and scientific methods that Nomad used to obtain this colicin preparation. Another advantage of using colicin or colicin-like bacteriocin is that colicin is highly sensitive to the action of protease enzymes, so any amount that would reach the human duodenum through food would be degraded in the intestines in a very short period [139].

### 5.2. Probiotic Activity

Probiotics are live microorganisms that may have health benefits to the host [140]. Often used in daily life, these microbes are mainly LAB, nonpathogenic *E. coli*, bacilli, or yeasts [141]. Probiotics bind to the intestinal epithelium and, thus, form a mechanical barrier that is necessary for the defensive immunity to prevent infections [142]. Specifically, the probiotic *E. coli* Nissle 1917 (EcN), under the name Mutaflor^®^, has been used in human and veterinary clinical treatments, to help in the treatment of intestinal disorders [143,144,145]. When EcN colonizes the gut, it induces epithelial cells to produce human β-defensin; this represents host immune response [146], promotes the secretion of microcins [147], and inhibits the growth and biofilm formation of other *E. coli* strains [148,149]. According to Fang et al. [150], probiotic *E. coli* Nissle 1917 can inhibit the biofilm formation of several pathogens, including enterohemorrhagic *E. coli* (EHEC), *Pseudomonas aeruginosa*, *Staphylococcus aureus*, and *S. epidermidis*. Probiotic *E. coli* have inhibitory effects on pathogenic biofilms via extracellular DegP activity during dual-species biofilm formation. EcN secretes DegP, a bifunctional (protease and chaperone) periplasmic protein and controls microbial biofilm formation; [150,151] showed that colicin Ib, E1, and microcin C7, derived from *E. coli* H22 have the ability to inhibit the growth of *Enterobacter, Escherichia, Klebsiella, Morganella, Salmonella, Shigella*, and *Yersinia*. These authors suggest its potential probiotic application.

## 6. Conclusions 

*Enterobacteriaceae* belong to the normal microbiota present in the gastrointestinal tract of humans and animals. Hence, these bacteria are constantly in contact with prokaryotic and eukaryotic cells in the GIT, which leads to the secretion of bacteriocins and biofilm formation, resulting in a very significant impact on gastrointestinal microniches. Moreover, *Enterobacteriaceae* can also be responsible for a variety of diseases and are frequently detected in foods, especially those produced from raw materials, being considered effective indicators of meat and dairy product quality and hygiene throughout the manufacture process. This manuscript presents an overview of the bacteriocins produced by *Enterobacteriaceae*, named colicins and microcins, the significance and impact of their interactions with prokaryotic and eukaryotic cells, as well as their putative use in food biotechnology and medicine. Overall, it becomes evident that there is a need for further research on this area of knowledge. Bearing in mind the urgent requirement for novel antimicrobials and anticancer agents, *Enterobacteriaceae*, and especially colicins and microcins, should be considered valid options.

## Data Availability

Not applicable.
